# Three dimensional printed polylactic acid-hydroxyapatite composite scaffolds for prefabricating vascularized tissue engineered bone: An *in vivo* bioreactor model

**DOI:** 10.1038/s41598-017-14923-7

**Published:** 2017-11-10

**Authors:** Haifeng Zhang, Xiyuan Mao, Danyang Zhao, Wenbo Jiang, Zijing Du, Qingfeng Li, Chaohua Jiang, Dong Han

**Affiliations:** 1 Department of Plastic and Reconstructive Surgery, Shanghai Ninth People’s Hospital, Shanghai Jiao Tong University School of Medicine, Shanghai, China; 2 Department of Plastic and Reconstructive Surgery, Renji Hospital, Shanghai Jiao Tong University School of Medicine, Shanghai, China; 3 Clinical Translational Research and Development Center of 3D Printing Technology, Shanghai Ninth People’s Hospital, Shanghai Jiao Tong University School of Medicine, Shanghai, China; 4 Shanghai Key Laboratory of Tissue Engineering, Shanghai Ninth People’s Hospital, Shanghai Jiao Tong University School of Medicine, Shanghai, China; 5 Shanghai Key Laboratory of Orthopaedic Implants, Shanghai Ninth People’s Hospital, Shanghai Jiao Tong University School of Medicine, Shanghai, China

## Abstract

The repair of large bone defects with complex geometries remains a major clinical challenge. Here, we explored the feasibility of fabricating polylactic acid-hydroxyapatite (PLA-HA) composite scaffolds. These scaffolds were constructed from vascularized tissue engineered bone using an *in vivo* bioreactor (IVB) strategy with three-dimensional printing technology. Specifically, a rabbit model was established to prefabricate vascularized tissue engineered bone in two groups. An experimental group (EG) was designed using a tibial periosteum capsule filled with 3D printed (3DP) PLA-HA composite scaffolds seeded with bone marrow stromal cells (BMSCs) and crossed with a vascular bundle. 3DP PLA-HA scaffolds were also combined with autologous BMSCs and transplanted to tibial periosteum without blood vessel as a control group (CG). After four and eight weeks, neovascularisation and bone tissues were analysed by studying related genes, micro-computed tomography (Micro-CT) and histological examinations between groups. The results showed that our method capably generated vascularized tissue engineered bone *in vivo*. Furthermore, we observed significant differences in neovascular and new viable bone formation in the two groups. In this study, we demonstrated the feasibility of generating large vascularized bone tissues *in vivo* with 3DP PLA-HA composite scaffolds.

## Introduction

The reconstruction of large size bone defects caused by tumour resection, trauma or congenital malformation remains a major clinical challenge in reconstructive, orthopaedic and craniofacial surgeries^1^. The lack of suitable donor bone tissue and complex geometries of bone tissue structure hamper successful repair. To generate large bone tissues with customized geometries for repair applications and aesthetic needs to preserve natural contours, numerous approaches have been explored for bone defect reconstruction^2,3^.

Autologous bone grafting is currently considered the gold standard for treating large bone defects because this type of graft contains the appropriate cell types, vasculature and matrix for promoting new bone formation. However, the harvesting of autografts can result in donor site morbidity and high infectious risks^4^. Alternatively, processed allogenic and xenogenic bone grafts have been considered. However, even these types of grafts have drawbacks, including disease transmission, potential immunoreaction and poor osteoconduction^5^. Although many strategies have been used, none have the ideal characteristics necessary for repairing large bone defects and defects with complex geometries^6^.

The limitations associated with current bone grafting options have prompted the development of additional methods for augmenting bone repair, particularly in cases of large volume bone defects. Bone tissue engineering (BTE), aimed to regenerate new biological bone tissue similar to native bone tissue, is a rapidly advancing discipline in the field of regenerative medicine and tissue engineering techniques^7^. Classical BTE has three essential elements: seed cells, growth factors and bioactive scaffolds. Frequently, BTE is manipulated under *in vitro* conditions, such as in an *in vitro* bioreactor. To date, although tremendous advances for the construction of tissue-engineered bone have been observed, BTE has not considered the functional factors of a true regenerative microenvironment^8^. As a result, limited clinical success has been reported in clinical therapeutic studies of *in vitro* BTE.

Recently, *in vivo* bioreactors (IVBs), which are designed to mimic *in vivo* microenvironments, have been explored for the repair of large bone defects for clinical applications by using an *in vivo* BTE approach^9^. *In vivo* bioreactors are chambers or scaffolds implanted in an ectopic site in the body where ossified tissue can be grown in the bioreactor in predefined geometries. Local vessels are recruited, and composite tissues can be harvested for later transfer^10^. Many studies, including our previous study, have prefabricated large vascularized tissue engineered bones and have shown the feasibility of this *in vivo* bioreactor approach^11–13^.

The treatment of large bone defects should consider the functional repair capabilities of prefabricated vascularized tissue engineered bone and aesthetics to maintain specific shapes. While the IVB approach cannot provide control over the final shape of the generated bone tissue, scaffolds can be designed with predefined geometries^14^ by using three dimensional printing techniques. Three dimensional printing is a rapid prototyping technique that can create complex 3D structures and provide unique ways to build accurate and highly reproducible scaffolds^15^. Specifically, three dimensional printed (3DP) scaffolds can be closely controlled and customized with proper shape, bioactivity, pore size and porosity for specific applications^16,17^.

Regarding the appropriate selection of bone substitute scaffolds for use in three dimensional printing techniques, many studies have presented different opinions. Among many bone graft substitutes, 3DP PLA-HA composite scaffolds have shown relatively good properties in biodegradability, biocompatibility and osteoconductivity^18^. Furthermore, PLA-HA composite scaffolds have been extensively applied in animal experiments and clinical studies^19,20^. However, no studies have indicated the use of three dimensional printing techniques with PLA-HA scaffolds in IVB strategies to generate vascularized tissue engineered bone of customizable size and geometry. The objectives of the present study were to observe whether vascularized bone tissue could regenerate by combining 3DP PLA-HA with an IVB and to analyse the regenerative level of vascularisation and osteogenesis and further to explore the feasibility of this approach for the prefabrication of vascularized bone tissue.

## Results

### Animal care and gross observation

After four and eight weeks of implantation, bioreactors were filled with 3DP scaffolds and successfully implanted in each animal. Implantation was well-tolerated in all animals with no infections, inflammation, or rejection.

### Quantitative detection genes expression

**Figure 1:**
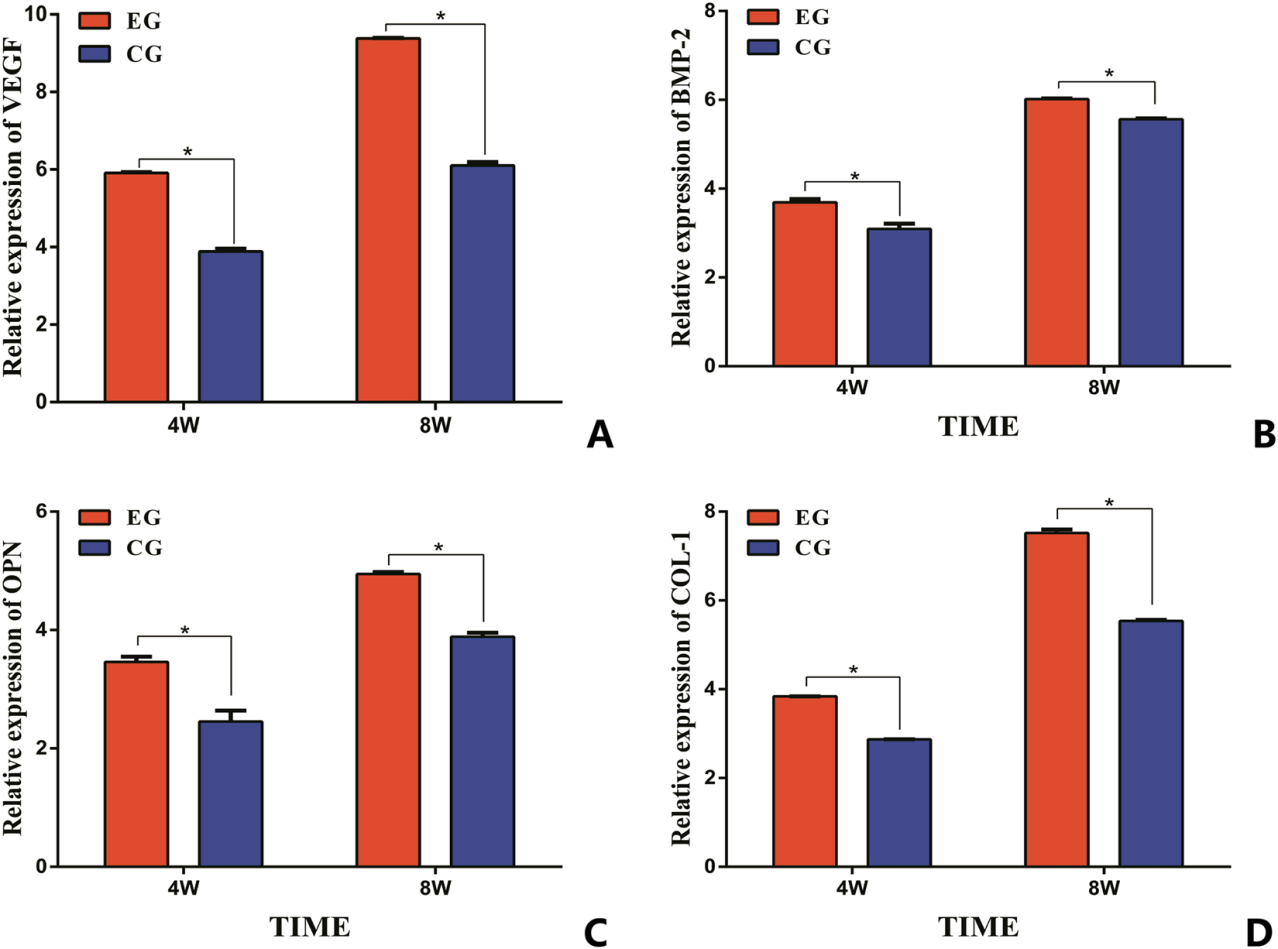
The expression of angiogenesis gene (VEGF (**A**) and BMP-2 (**B**)) and osteogenic gene expression (OPN (**C**) and COL-1 (**D**)) in two groups. (*p < 0.05).

To better characterize the expression of angiogenesis and osteogenic biomarkers generated within the *in vivo* bioreactors, qRT-PCR was performed for VEGF and BMP-2 expressed during vascularisation and for OPN and COL-1, indicative of viable bone formation. As shown in Fig. 1, four genes related to angiogenesis and osteogenic were detected. The experimental group (EG) revealed significantly greater expression of all four genes compared with the control group (CG). Significant differences were observed between the two groups at 4 and 8 weeks after implantation (P < 0.05).

### Micro-CT Measurement

**Figure 2:**
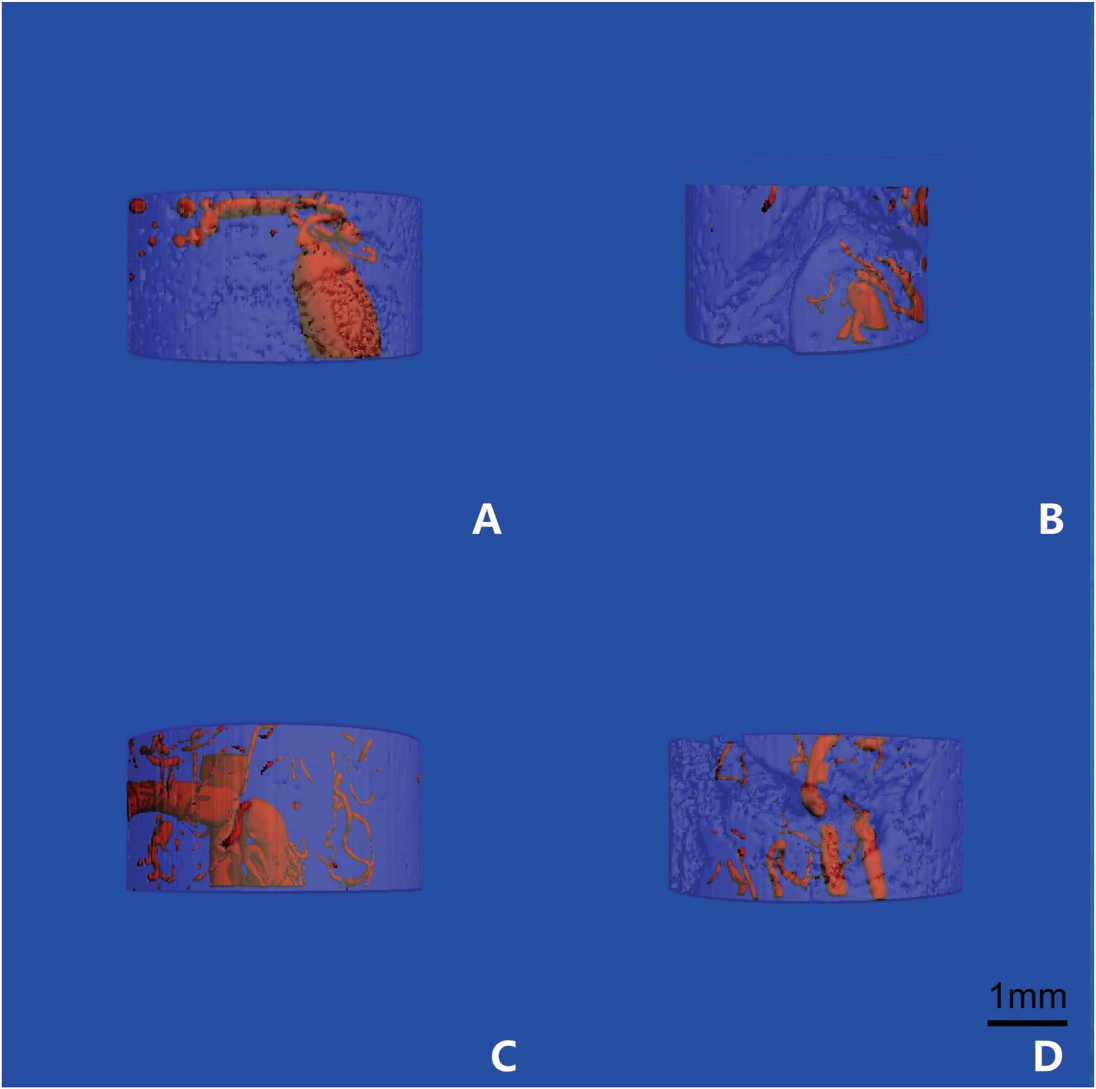
The architecture of microvascular was scanned by Micro-CT. The structures of angiogenesis in EG (**A**) and in CG (**B**) at 4 weeks *in vivo*; the structures of angiogenesis in EG (**C**) and in CG (**D**) at 8 weeks *in vivo*.

**Figure 3:**
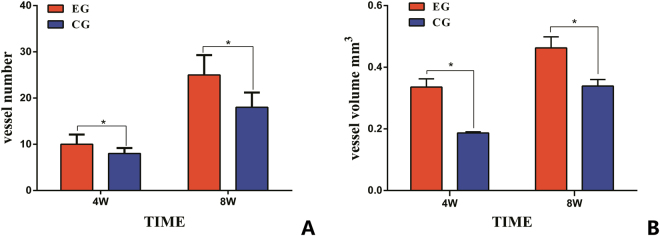
Micro-CT quantification of new vessel formation in 3DP PLA-HA composite scaffolds between two groups. (**A**) Mean numbers of blood vessels were compared in two groups after 4 and 8 weeks of implantation. (**B**) The average total volume of new vessel was compared in two groups after 4 and 8 weeks of implantation. (*p < 0.05).

The structures of microvasculature and newly formed bone tissues in bioreactor specimens were examined using microangiography and micro-CT. As shown in Fig. 2, microvascular architecture and angiogenesis could be observed in the two groups. Newly formed vessels were shown growing throughout the *in vivo* bioreactor and were found inside and outside the scaffold samples. 3D reconstructions of the micro-CT scans demonstrated that the average number of vessels and the average total vessel volume penetrating the *in vivo* bioreactor were higher in the EG than in the CG (Fig. 3). The number and volume of blood vessels increased at 8 weeks compared with at 4 weeks after implantation. The differences between the experimental and control samples were significantly different (p < 0.05, respectively).

**Figure 4:**
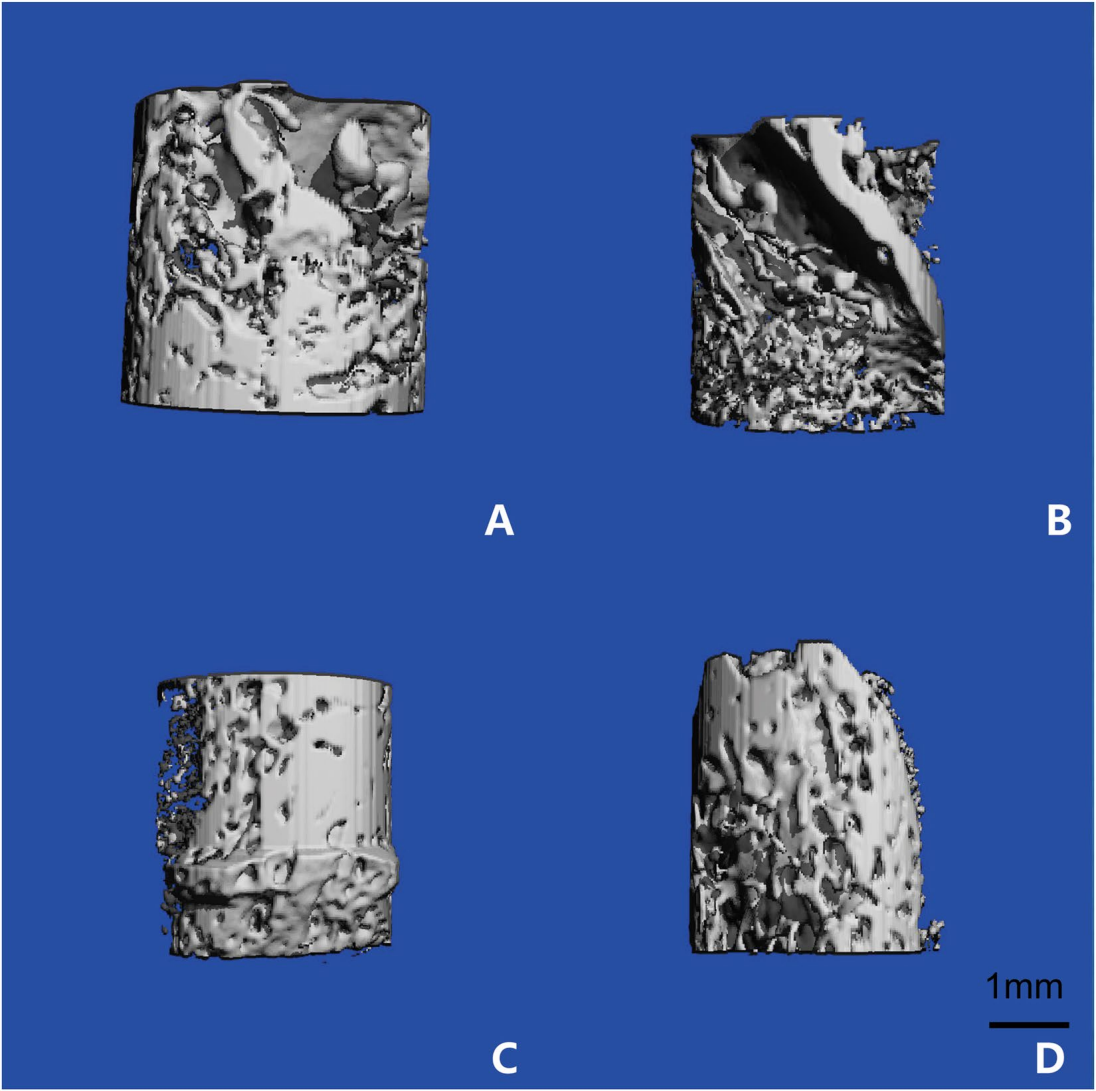
The structure of the newly formed bone was scanned by micro-CT. The formation of neo-osseous tissue in EG (**A**) and in CG (**B**) at 4 weeks after implantation; the formation of neo-osseous tissue in EG (**C**) and in CG (**D**) at 8 weeks after implantation.

**Figure 5:**
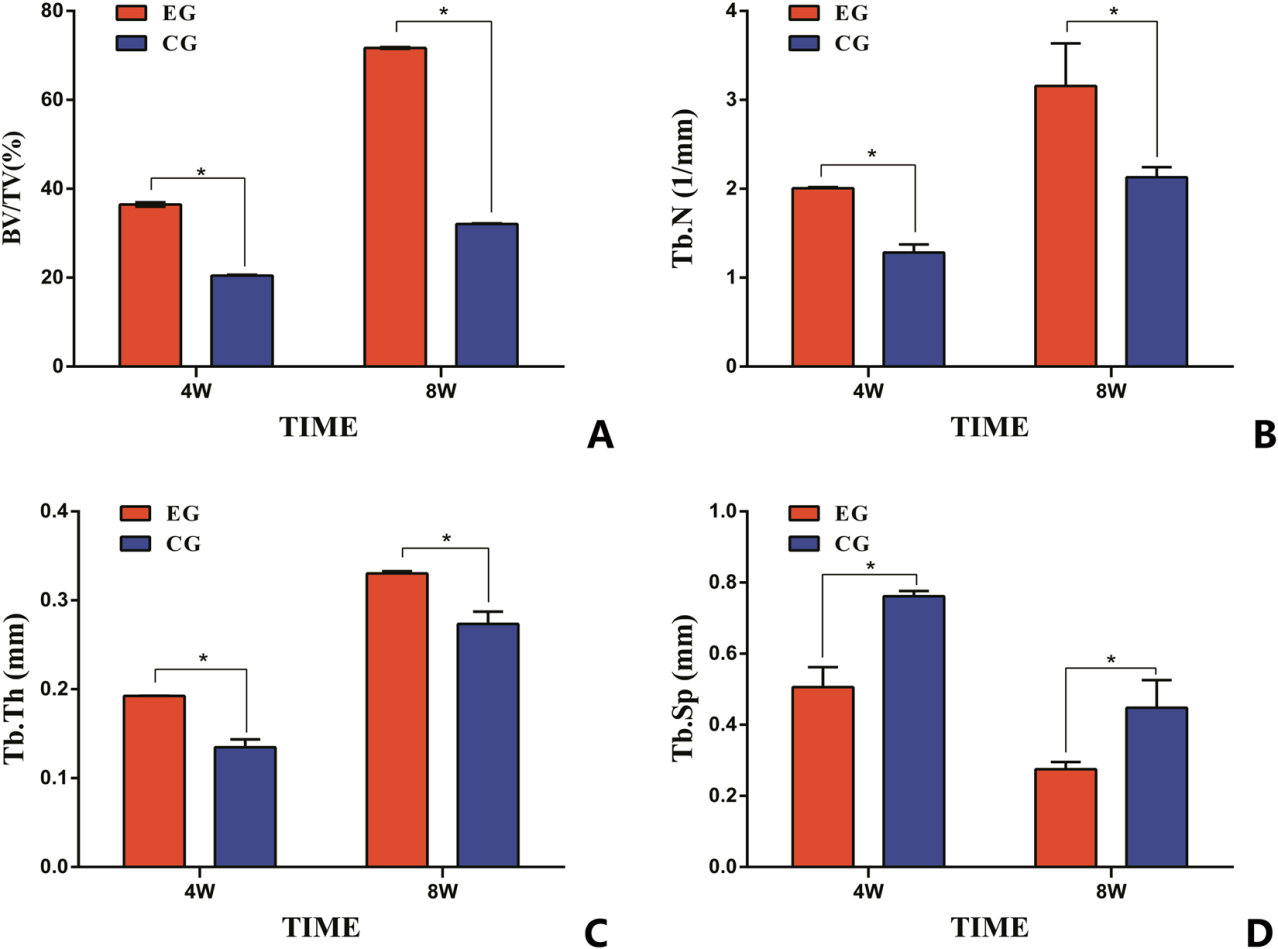
Micro-CT detection of osteogenesis within the constructs after 4 and 8 weeks of *in vivo* implantation. (**A**) Quantitative morphometric analysis results of BV/TV; (**B**) Quantitative morphometric analysis results of Tb.N; (**C**) Quantitative morphometric analysis results of Tb.Th; (**D**) Quantitative morphometric analysis results of Tb.Sp. (*p < 0.05).

From Fig. 4, newly formed bone tissue and a strengthened ossification trend could be observed at different time points. The architecture of the generated bone tissue was assessed, and the values of BV/TV, Tb.N and Tb.Th were significantly greater for the EG compared with the CG at 4 and 8 weeks after surgery. Tb.Sp values decreased in the EG compared with the CG. The differences between the experimental and control samples *in vivo* bioreactor were significantly different (p < 0.05, respectively) (Fig. 5).

### Histological analysish

**Figure 6:**
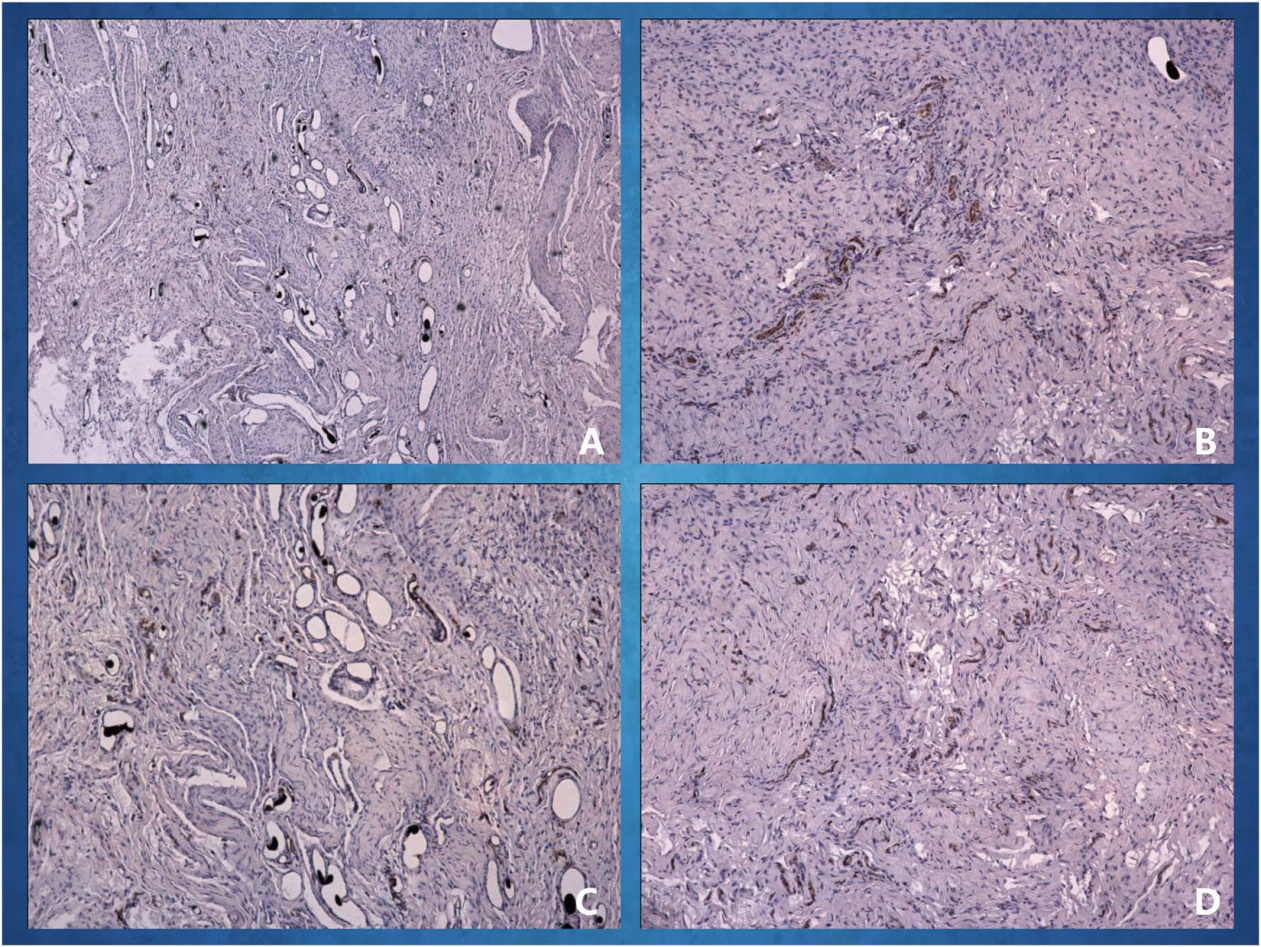
Immunohistochemical examination of CD31 was carried out at different times. The CD31 expression images of the *in vivo* bioreactor by implanting 3DP PLA-HA in EG (**A**) and in CG (**B**) after 4 weeks of implantation; The CD31 expression images in EG (**C**) and in CG (**D**) after 8 weeks of implantation.

**Figure 7:**
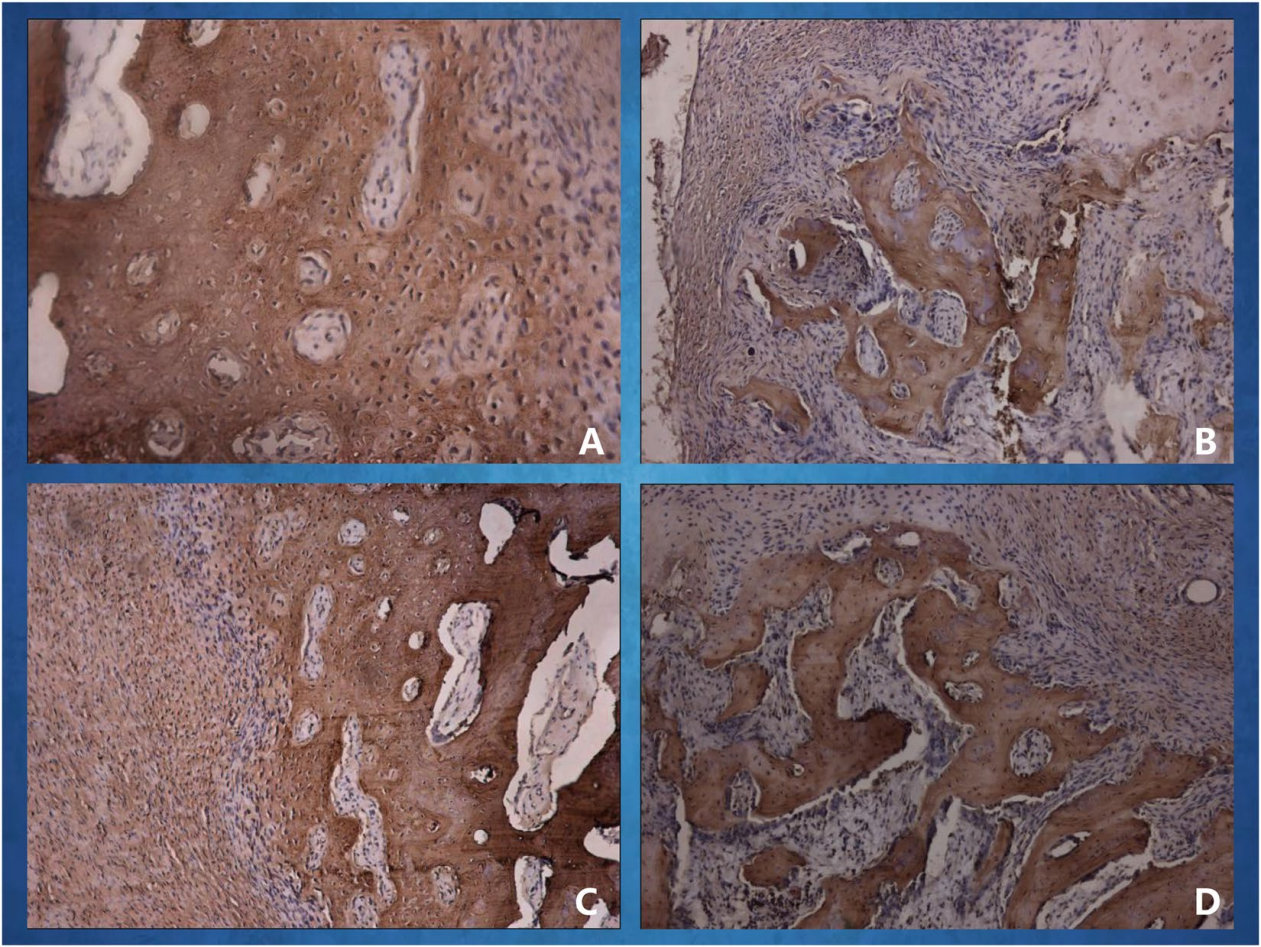
Immunohistochemical examination of osteocalcin (OCN) was carried out at different times. The OCN expression images *in vivo* bioreactor by implanting 3DP PLA-HA in EG (**A**) and in CG (**B**) at 4 weeks after implantation; the OCN expression images in EG (**C**) and in CG (**D**) at 8 weeks after implantation.

A histological examination of the specimens in the *in vivo* bioreactor revealed neovascular formation and ossified tissue generation in the two groups after 4 and 8 weeks of implantation in rabbits. As shown in Fig. 6, newly formed blood vessels were clearly observed in the EG; these few blood vessels were seen inside the bone tissue with infiltrating fibrous connective tissue. As shown in Fig. 7, new viable bone formation was demonstrated in the two groups. The lacunae of the neo-tissue, which were easily identified as osseous tissue, can be further visualized in the EG, whereas some areas of less mature woven bone within the neo-tissue were seen in the CG. Compared with at 4 weeks after implantation, at 8 weeks, stains of new blood vessels and bone tissue were more intense and uniform indicating the development of blood vessel distribution and mature osseous tissue.

**Figure 8:**
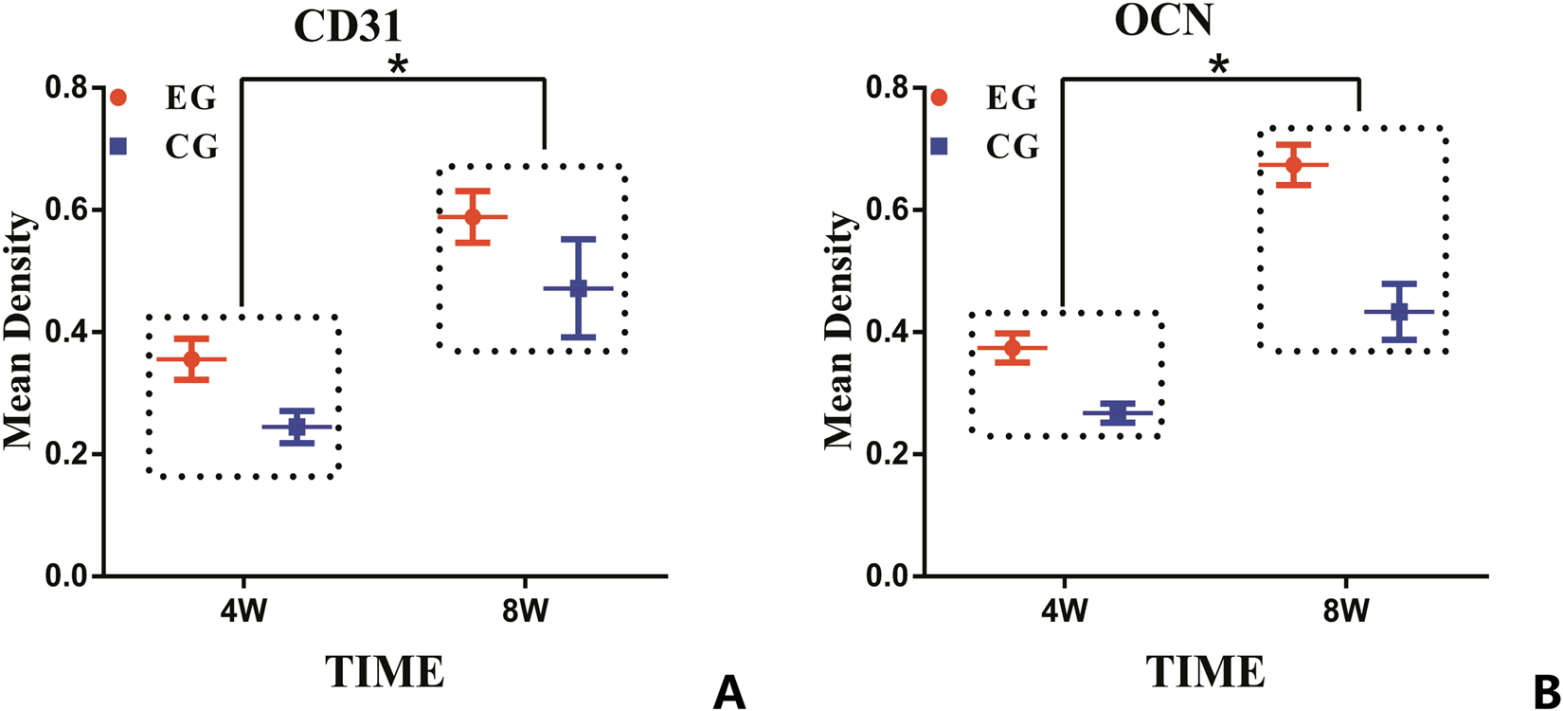
The semi-quantitative scatter plot of CD31 and osteocalcin (OCN) expression. (**A**) The CD31 expression images in two groups at 4 and 8 weeks after implantation. (**B**) The OCN expression images in two groups at 4 and 8 weeks after implantation. (*p < 0.05).

Immunohistochemical analyses were performed to provide a more detailed analysis of the tissue formation process. The mean amounts and percentages of blood vessels and bone tissue positively stained for CD31 and OCN were higher in the EG compared with the CG. At 8 weeks after implantation compared with at 4 weeks, there were higher positive expression levels of CD31 in enhanced capillary formation and of OCN in new bone formation (Fig. 8). The differences between the experimental and control samples were significant (P < 0.05, respectively). These results correlated with the results determined by the micro-CT analysis.

## Discussion

The reconstruction of large volume bony defects with customizable dimensions remains a significant clinical challenge. To generate vascularized large bone tissue as ideal functional bone grafts and to shape them as best as possible for aesthetic purposes, many studies have explored various reconstruction methods and biomaterial manufacturing techniques^21–24^. Rapid advances in bioreactor *in vivo* technology, tissue engineering practices, and 3D printing technologies represent promising strategies for bone defect reconstruction for reforming bones with shapes conforming to the geometry of the defect site.

A reason for poor bone formation may be insufficient vascularization of the scaffolds^25^. Vascularization is an essential prerequisite for bone development and for the regeneration of osteogenic cells. Furthermore, vascularization provides enhanced transport for oxygen, nutrients, and important bioactive factors to bone formation areas^26,27^. When compared with in vitro bone tissue engineering, these *in vivo* bioreactors more readily promote angiogenesis. As previously described^11^, to generate extensive vascularization and osteogenesis in the scaffolds, a saphenous vascular bundle was combined with periosteum. To prefabricate a vascularized bone graft, a saphenous vascular bundle was centrally inserted into a scaffold to provide transport for progenitor cells, cytokines, oxygen and nutrients and to remove waste products from cells^28^. The periosteum is a thin but highly vascularized tissue that provides a number of osteoblasts, pluripotent stem cells, and progenitor cells for vascularized bone growth^29^. A periosteal flap can be used to wrap the tissue-engineered construct to minimize tissue disturbance^30^. For these reasons, the combined use of a blood vessel and periosteum can take advantage of two well-established bone graft prefabrication strategies.

Seeding cells, growth factors and bioactive scaffolds are three essential elements for bone tissue engineering. Many approaches have involved various combinations of these elements to regenerate vascularized bone tissue^31^. However, conflicting studies have indicated contradictory uses of exogenous growth factors as core components for in vitro tissue engineering. In our study, BMSCs were selected as seed cells, whereas angioinductive or osteoblast growth factors were not used. Many studies have demonstrated that BMSCs can be used for *in vivo* graft colonization to enhance the effectiveness of bone formation^32,33^. Regarding the introduction of exogenous growth factors to *in vivo* bioreactors, a few studies have suggested that these growth factors carry risks of tissue overgrowth and nerve impingement and may increase the complexity of the technology and associated regulatory burdens for clinical translation^34,35^. Thus, strategies utilizing *in vivo* bioreactors without exogenous growth factors are being explored. This represents a major advancement in the field and facilitates clinical translation.

To support viable bone tissue growth, *in vivo* bioreactors must be filled with appropriate scaffold materials^36^. Over the past decade, scaffold design remains the main challenge in tissue engineering due to the large number of requirements that need to be met, such as having the ability to deliver cells, supporting differentiation of regenerative cells, irregular geometries, biocompatibility, osteoconductivity and osteoinductivity^37,38^. To achieve geometric precision of the bone construct to conform to the contours of the recipient site, the fabrication of custom-contoured scaffolds is critical. With the aid of three-dimensional printing technology, the geometry and material properties of scaffolds can be controlled and tailored to the shape of defects^39^.

Among the various materials used in recent years, PLA-HA scaffolds have demonstrated their ability to promote new bone regeneration^40,41^. PLA is a polymer of lactic acid with high biocompatibility and degradability and can be easily fabricated into porous scaffolds^42^. HA can act as a reinforcing material to improve the osteoconductivity of the scaffold^43^. PLA and HA have been mixed to form a porous scaffold that exhibits the superimposed behaviour of ceramics in a polymeric matrix^19^. PLA-HA composite materials have been formed as scaffolds using three-dimensional printing technology and have been shown to have good biocompatibility and bioactivity *in vitro* ^18^. In the present study, the good osteogenic capability and osteoinductive activity of a 3DP PLA-HA *in vivo* bioreactor were estimated and were shown to enhance bone formation.

*In vivo* bioreactors have been successfully used to create a series of complex tissues. Various types of scaffolds, orthotopic sites and vascular bundles have been considered for the vascularization and remodelling of regenerated bone tissue^28^. Studies have described *in vivo* bioreactors using vascular bundles filled with different scaffold materials. Holt *et al*. described an effective *in vivo* bioreactor using a coral cylinder scaffold implanted within the superficial inferior epigastric artery and vein^44^. Kokemueller and colleagues used a muscular pouch in the latissimus dorsi muscle by combining an axial vascular bundle from the thoracodorsal trunk with β-TCP and iliac crest autograft scaffolds to prefabricate bone grafts to repair mandibular defects^45^. Kloeters *et al*. applied processed bovine cancellous bone penetrated by a vascular bundle from inguinal vessels for the vascularization and osteogenesis of a prefabricated bone graft^46^. Our previous research reported an *in vivo* bioreactor that used a saphenous vascular bundle threaded through beta tricalcium phosphate (β-TCP) granules wrapped by muscularis membrane^23^. However, in the present study, for the first time, we prefabricated vascularized bone grafts by inserting a saphenous vascular bundle into 3DP PLA-HA composite materials wrapped by a periosteal flap. The results showed a developed capillary network and new bone tissue at regenerated sites.

The mechanical stress is a critical property for estimating the strength of vascularized bone tissue. While there is a lack of detailed evidence on new bone formation due to mechanical stress, the micro-CT results in our study determined the mechanical strength of new bone tissue. We found that in the EG, the average values of BV/TV and Tb.N and Tb.Th were greater and that of Tb.Sp was smaller than those in the CG. According to Mittra, the measured micro-CT parameters and mechanical strengths of BV/TV, Tb.N, and Tb.Th were found to be positively correlated with increasing mechanical properties, whereas Tb.Sp was negatively correlated with increasing mechanical properties^47^. Therefore, the outcomes suggested that bone tissues grown within the *in vivo* bioreactor in the EG had greater mechanical strength than that in the CG. In future studies, additional mechanical analyses will be performed to further explore the nature of generated bone tissue.

Our study indicated that *in vivo* bioreactors and 3DP PLA-HA composite scaffolds are promising tools for the prefabrication of vascularized tissue engineered bone. We showed that such tools were sufficient to generate bone tissue capable of integrating with native tissues when transferred to a large bone defect. The use of these tissues in the reconstruction of large bone defects requires further study. Additional studies will need to be performed to further elucidate the mechanisms governing neovascularization and osteogenesis within *in vivo* bioreactors.

## Conclusions

We demonstrated that large volume, customized, vascularized bone tissues can be prefabricated using 3DP PLA-HA composite scaffolds in an *in vivo* bioreactor without requiring the use of exogenous growth factors. Compared with *in vivo* bioreactors only implanted in the periosteum, the addition of a vascular bundle has a greater advantage in constructing large vascularized bone grafts. With reliable microsurgical transplantation, this novel approach may be a bridge for repairing large bone defects and facilitating clinical translation in bone tissue engineering.

## Materials and Methods

### Fabrication of the biomaterials

**Figure 9:**
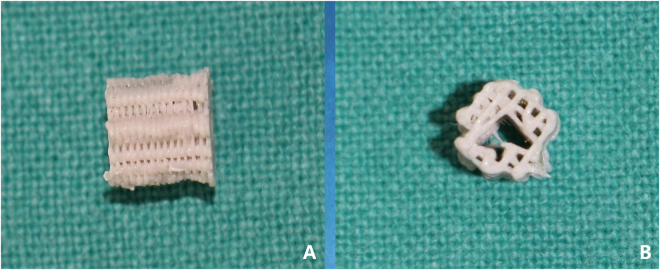
3D printed PLA-HA composite scaffolds were cylindrical with a central channel. (**A**) Front view; (**B**) lateral view.

Poly(L-lactide) (PLA) was purchased from Sigma-Aldrich (Shanghai, China), and hydroxyapatite (Ca _10_(PO_4_)_6_(OH)_2_) (HA) was purchased from Sonac Company (The Netherlands) with a mean size of 2.1 ± 0.4 μm. As previously described, a new mini-deposition system (MDS) located in the Shanghai 3D printing centre was developed to fabricate scaffolds. PLA (85 wt.%) and HA (15 wt.%) were mixed and processed to produce PLA-HA composite scaffolds with the MDS method^48,49^. Based on three-dimensional computed tomography reconstruction, computer-aided design and computer-aided manufacturing systems, the 3D printer produced porous cylindrical scaffolds with a predetermined structure (5-mm diameter, 6-mm height; Fig. 9). These scaffolds with predefined configurations had a mean pore size of 500 μm and an average porosity was 60%. The 3D printed scaffolds were sterilized by ethylene oxide and prepared for study.

### Animals

**Figure 10:**
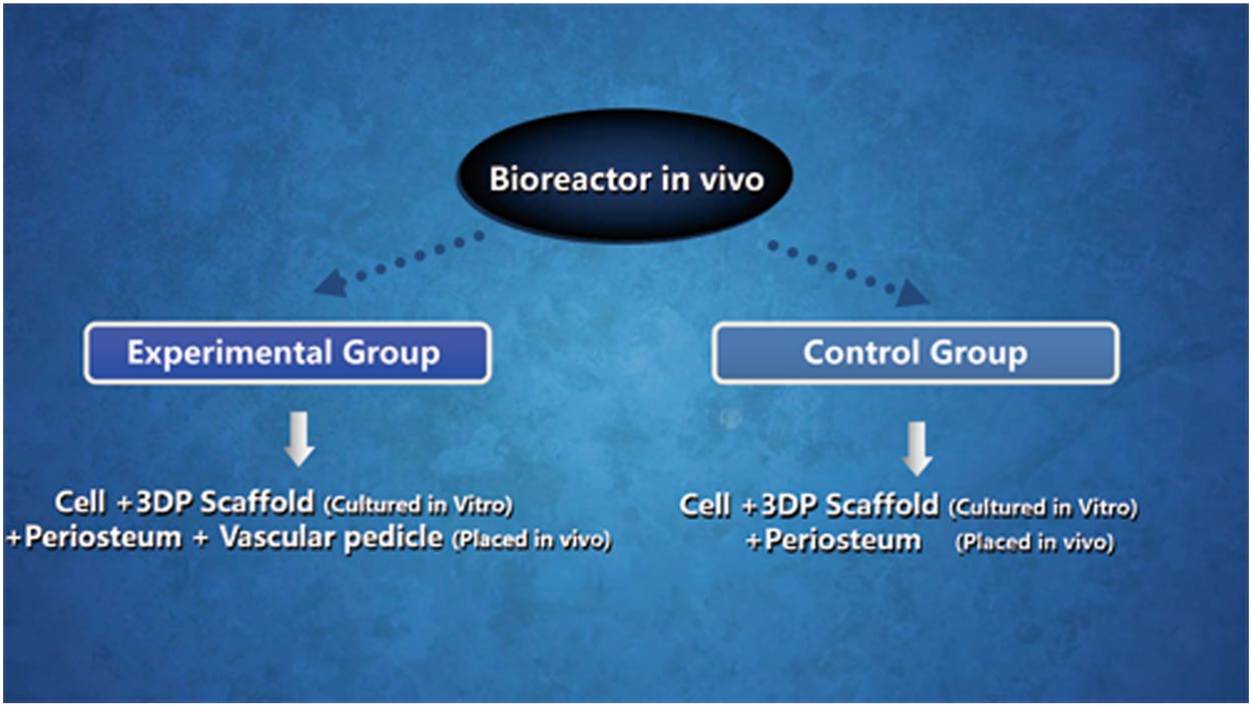
The experimental design in two groups including the experimental group (EG) and control group (CG).

**Figure 11:**
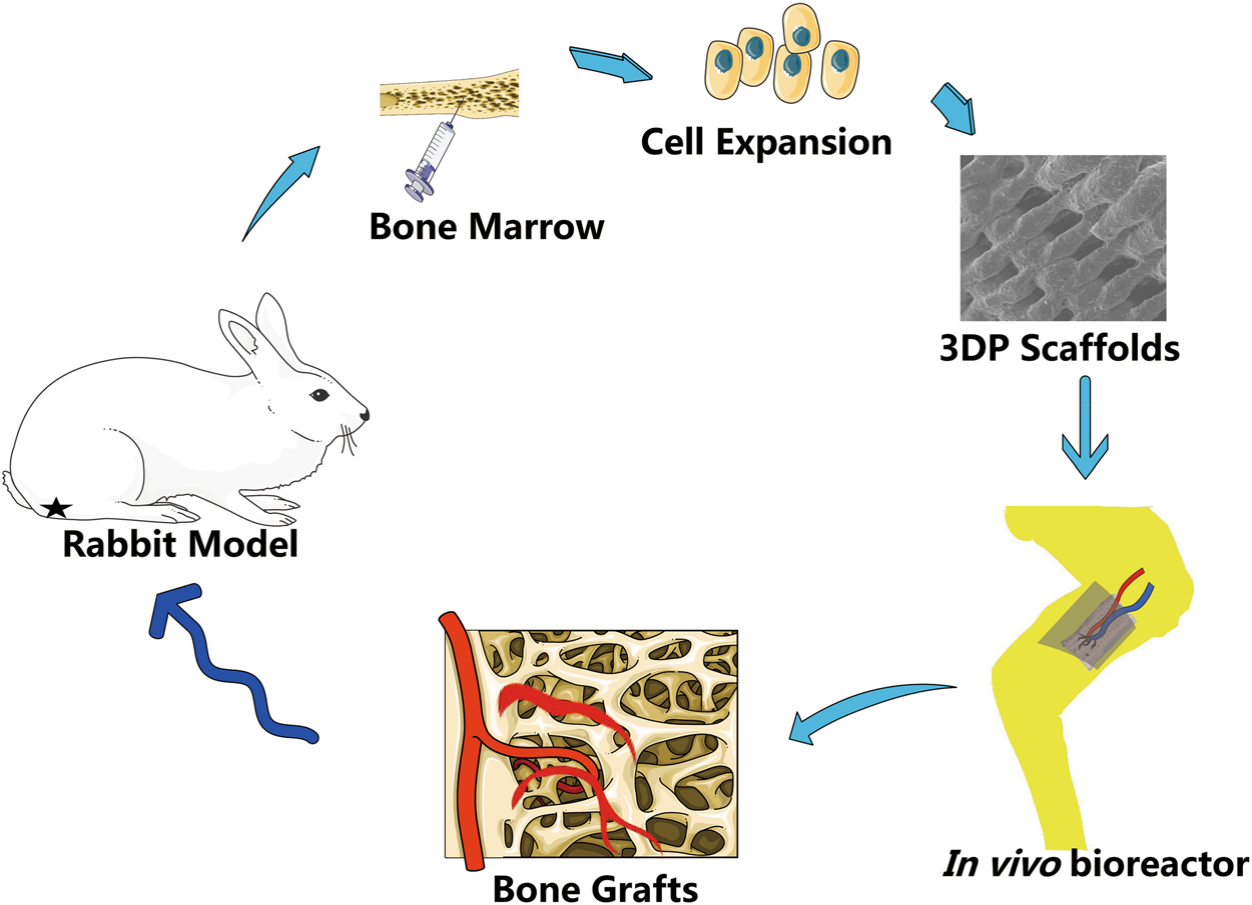
The simplified diagram of experimental procedures in our study.

Twenty-four New Zealand white adult rabbits (6 months of age, ranging from 2.5 ± 0.2 kg in weight) were used in accordance with the guidelines of the Institutional Animal Experiment Department of Shanghai Jiao Tong University (Shanghai, China) and the principles of laboratory animal care (NIH publication number 85–23, revised 1985). The ethics committee of Shanghai Jiao Tong University specifically approved this study. All rabbit groups included one experimental group (EG) and one control group (CG) (Fig. 10). The general operating procedures are shown in Fig. 11. For each group, after implantation, six rabbits were euthanised at 4 and 8 weeks.

### *In vitro* 3DP scaffolds combined with bone marrow stromal cells (BMSCs)

Bone marrow stromal cells (BMSCs) were harvested from New Zealand white adult rabbits. Bone marrow aspirates (2 mL) were taken from the lateral tibial tubercle using 5-mL injectors with 0.5 mL heparin sodium (2500 U/mL, Wanbang, China Chemical & Pharmaceutical Company, Jiangshu, China). The number of bone marrow aspirates was the same as the number of rabbits. Bone marrow was flushed with 20 mL Dulbecco’s minimum Essential medium (DMEM; Gibco, Australia) supplemented with 10% foetal bovine serum (FBS; HyClone; America), penicillin (50 U/ml) and streptomycin (50 mg/ml).The mixture was centrifuged at 1000 rpm for 5 min, and the supernatant was removed. The cell pellet was re-suspended in DMEM and directly transferred to a 10-cm culture dish at 37 °C in an atmosphere of 5% CO_2_. The medium was exchanged twice a week. Before implanting BMSCs, 3DP scaffolds were transferred into 24-well cell culture plates and incubated with culture medium for 24 h. BMSCs were collected and suspended at a density of 5 × 10^6^/mL. BMSCs were seeded on each scaffold in a 24-well plate and allowed to adhere to the scaffold for 4 h to permeate into the material. Then, 10 mL DMEM was added.

### Implantation of the *in vivo* bioreactor

**Figure 12:**
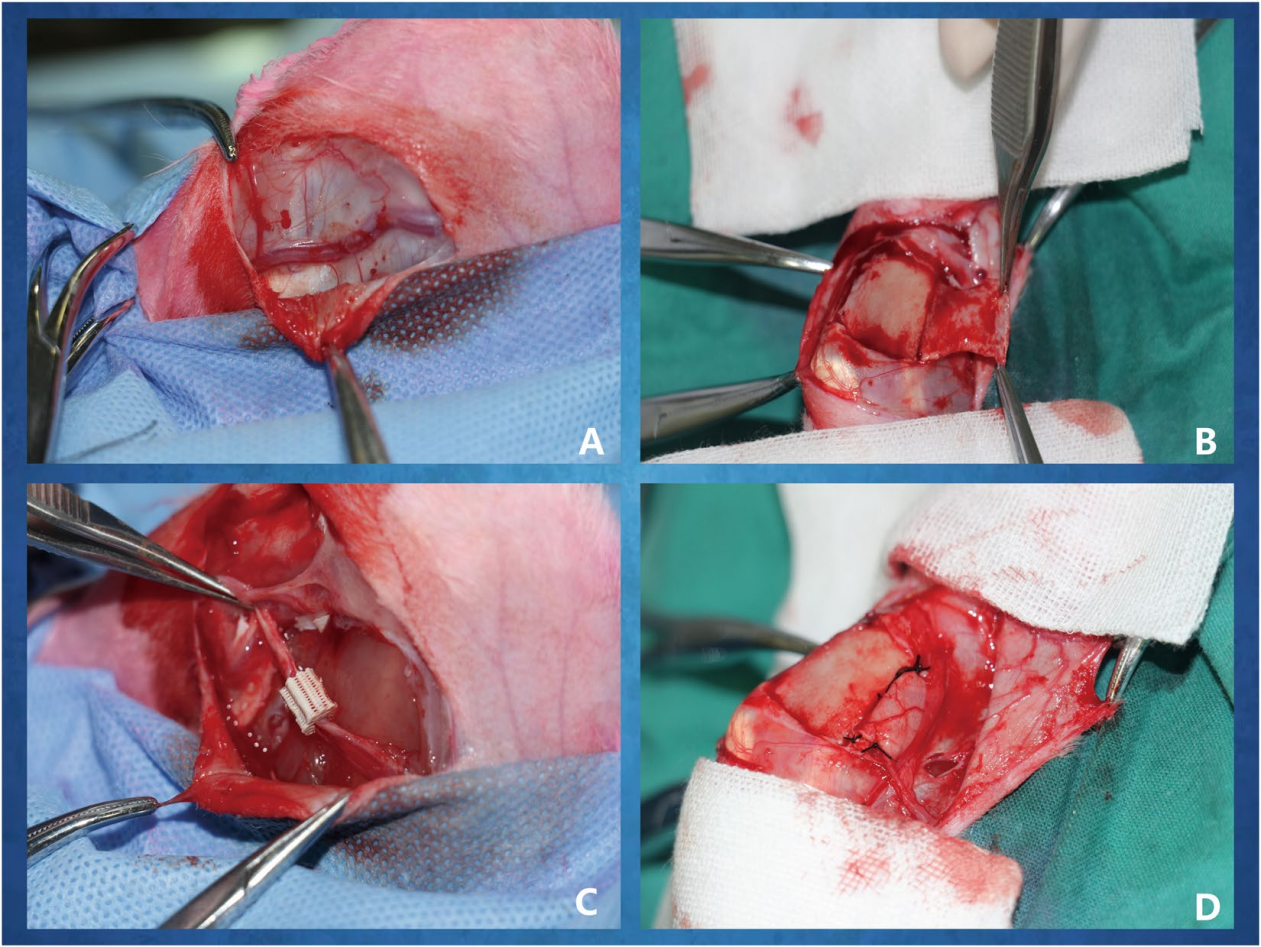
Surgical procedures to finish the bioreactor implantation *in vivo*. (**A**) The saphenous arteriovenous blood bundles were fully free; (**B**) the periosteum was elevated on the surface of the tibia; (**C**) the saphenous vessel bundle was crossed through the central channel of 3D printed PLA-HA composite scaffolds combined with autologous BMSCs; (**D**) the scaffolds were rolled with the pedicle-attached periosteum to construct a periosteum capsule.

All New Zealand white rabbits were randomly divided into two groups, and an animal model of an *in vivo* bioreactor on adult New Zealand rabbits inside the tibia was established. Bioreactor implantation was performed with established methods^11,50,51^. Briefly, animals were anaesthetized and intubated. Under sterile operating conditions, a 4-cm incision was made on the medial aspect of the tibia of the hind legs of the rabbits. Skin and underlying subcutaneous tissue were elevated and extended. Then, a vessel bundle, including a saphenous artery and a saphenous vein, was completely exposed (Fig. 12A). The saphenous arteriovenous blood bundles were fully separated. Distal ends of the pedicles were tied and ligated, and the proximal portion of the pedicle remained attached to the circulatory. The periosteum on the surface of the tibia was elevated and isolated (Fig. 12B). This operation should ensure the preservation of blood flow to the periosteum. Cuboid-shaped periosteal pockets 10 mm in length and 7.5 mm in diameter were created. Then, the pedicles were threaded through the central core of the cylindrical scaffolds and combined with autologous BMSCs (Fig. 12C) and secured by tying sutures under the periosteum capsule (Fig. 12D). After finishing all operations, vascular blood flow of the pedicles was confirmed (Video 1). An *in vivo* bioreactor in the experimental group (EG) was surgically inserted by the above operation, and an avascular bioreactor was designated the control group (CG). The soft tissue was sutured with 6–0 PDS sutures, and the skin was closed with a 5–0 PDS suture. The animals were returned to their cages and monitored post-operatively for signs of discomfort or adverse events. The rabbits were euthanised at 4 and 8 weeks, and the specimens were harvested.

### Microfil perfusion

To demonstrate and visualize new blood vessel formation, as described by Bolland^52^, the lower abdominal aorta was perfused with Microfil (Flowtech, Carver, MA, USA) following euthanasia at 4 and 8 weeks post-operative. Briefly, a midline incision extending across the abdomen was made, and the lower part of the abdominal aorta was exposed. Then, 30 mL Microfil were perfused at a steady constant flow after clamping the upper part of the abdominal aorta. *In vivo* bioreactor-generated tissues were harvested from animals at 4 °C overnight. The tissues were stored in 10% neutral buffered formalin (NBF) for subsequent experiments.

### RNA isolation and qRT-PCR

Quantitative real-time reverse transcription polymerase chain reaction (qRT-PCR) was used to measure vascularisation and osteogenic differentiation. To determine angiogenesis and osteogenic gene expression levels, mRNA was isolated from biopsies surrounding the tissues generated in the *in vivo* bioreactors.

Vascular endothelial growth factor (VEGF) and bone morphogenetic protein-2 (BMP-2) were genes of interest because they are important regulators of angiogenesis and stimulate new vessel formation^53,54^. Osteogenic-related genes, osteopontin (OPN) and collagen type I (COL-1), were selected to estimate the levels of osteogenesis, respectively. QRT-PCR was performed on 3-mm biopsy samples. To recover and isolate mRNA, biopsy specimens were flash frozen in liquid nitrogen, crushed, and placed in TRIzol reagent (Invitrogen Pty, Ltd., Australia). mRNA was subsequently reverse-transcribed into cDNA using a PrimeScript^TM^ RT reagent kit (Takara, Japan). Then, quantitative PCR was then performed with forward and reverse primers using SYBR Green detection reagent. The final analysis was performed on a 7500 Real Time PCR System (Applied Biosystems, USA). The relative expressions of the genes of interest were normalized against housekeeping gene glyceraldehyde 3-phosphate dehydrogenase (GAPDH)^55^. As designed from PubMed, the forward and reverse primer sequences were as follows. For VEGF: forward 5′-CGA ACG TAC TTG CAG ATG TGA C-3′ and reverse 5′-CAA AGT GCT CAC GCA GTC TC-3′; for BMP-2: forward 5′-TTT GGT CAC GAT GGG AAG GG-3′ and reverse 5′-TGC ACG ATG GCA TGG TTA GT-3′; for OPN: forward 5′-GTG TAC CCC ACT GAG GAT GC-3′ and reverse 5′-CAC GTG TGA GCT GAG GTC TT-3′; for COL-1: forward 5′-TCG ATC CCA ACC AAG GAT GC-3′ and reverse 5′-CAA ACT GGG TGC CAC CAT TG-3′; and for GAPDH: forward 5′-TGG AAT CCA CTG GCG TCT TC-3′ and reverse 5′-GTC ATG AGC CCC TCC ACA AT-3′. The mean cycle threshold (Ct) value of each target gene was normalized against the Ct value of GAPDH. The relative expression level of each target gene was evaluated using the 2^−(normalized average Ct)^ ×10 method^56^.

### Micro-computed tomography (micro-CT) analysis

All specimens were scanned using micro-CT (SCANCO MEDICAL AG, Switzerland) with a resolution of 10 μm to evaluate neurovascular and new bone formation. Then, 3D images were reconstructed using 3D Creator software (GE Healthcare BioSciences, Chalfont-St. Giles, UK). Blood vessels and bone tissues were separately reconstructed according to different grey value ranges. A region corresponding to vessels perfused with microfil on the micro-CT grey scale range was selected, and the parameters of vessel number (VN) and vessel volume (VV) were analysed. Three-dimensional measurements of the amounts of bone volume per total volume (BV/TV), trabecular number (Tb.N), trabecular thickness (Tb.Th) and trabecular spacing (Tb.Sp) in the bone tissue were also calculated by microCT.

### Histological observation

All harvested tissues were stored in 10% neutral buffered formalin (NBF) and decalcified in 10% ethylenediaminetetraacetic acid (EDTA) for 4 weeks at 37 °C prior to paraffin embedding. The tissue specimens were sliced into 10-μm sections along the long axis of the tissue by a microtome with a diamond blade. Immunohistochemical staining was carried out to assess and analyse the formation of new blood vessel and new bone tissue, respectively. The sections were incubated with primary antibodies against CD31 and osteocalcin (OCN; Abcam, Cambridge, MA, USA) at 4 °C overnight. After rinsing with PBS buffer, HRP-conjugated secondary antibody and DAB solution were used to stain the sections. Then, cell nuclei were identified with haematoxylin. Images were observed by light microscopy (TE2000U, Nikon, Japan) and were acquired with BioQuant OSTEO II software (BioQuant Image Analysis Corporation, Nashville, TN). The CD31 and OCN-positive area was assessed using Image ProPlus 6.0 software to evaluate angiogenesis and osteogenesis.

### Statistical analysis

The data are presented as the mean ± standard deviation. The normal distribution of all the data was proved by shapiro wilk test. Significant differences between the experimental and control bioreactors in each animal were examined with Student’s t-test analysis. SPSS 17.0 software was employed for all statistical analyses, and P < 0.05 was considered significant.

## Electronic supplementary material

video 1
